# Comparative drought resistance of temperate grassland species: testing performance trade-offs and the relation to distribution

**DOI:** 10.1007/s00442-020-04625-9

**Published:** 2020-02-29

**Authors:** Eun-Young Jung, Julian Gaviria, Shanwen Sun, Bettina M. J. Engelbrecht

**Affiliations:** 1 Department of Plant Ecology, Bayreuth Center of Ecology and Environmental Research (BayCEER), University of Bayreuth, 95440 Bayreuth, Germany; 2 Smithsonian Tropical Research Institute, Apartado 0843-03092, Balboa, Ancon, Republic of Panama

**Keywords:** Drought tolerance, Rainfall niche, Distribution patterns, Dryness, Climate change

## Abstract

To improve projections of consequences of increasing intensity and frequency of drought events for grasslands, we need a thorough understanding of species performance responses to drought, of performance trade-offs and how drought resistance is related to species distributions. However, comparative and quantitative assessments of whole-plant drought resistance that allow to rigorously address these aspects are lacking for temperate grassland species. We conducted a common garden experiment with 40 common temperate grassland species to compare species survival and growth under intense drought and well-irrigated conditions. Overall, survival and growth were significantly reduced under drought, with the effect varying across species. Species ranking of drought damage and survival remained consistent with progressing drought. No performance trade-offs emerged between optimal growth and drought resistance of survival (‘growth–stress tolerance’ trade-off hypothesis), or between growth under well-watered and dry conditions (‘growth rates’ trade-off hypothesis). Species local- and large-scale association with moisture (Ellenberg *F* value and rainfall niche) was not related to their drought resistance. Overall, our results imply that trade-offs and differences of species fundamental drought resistance are not the main drivers of hydrological niche differentiation, species coexistence and their distribution across moisture gradients. The comparative experimental assessment of species whole-plant drought responses we present provides a basis to increase our understanding of current grassland responses to variation of moisture regimes and for projecting consequences of future changes.

**Electronic supplementary material:**

The online version of this article (10.1007/s00442-020-04625-9) contains supplementary material, which is available to authorized users.

## Introduction

Drought is an important driver of community composition, diversity, and ecosystem function in a variety of ecosystems worldwide. Temperate grasslands are among the most widespread biomes on earth, exhibit high species richness, contain economically important species, and provide key ecosystem services (Gibson 2009). In a wide range of temperate grasslands, drought decreases productivity and influences species abundance and distribution, as well as community composition and diversity patterns (Tilman and El Haddi 1992; Buckland et al. 1997; Knapp et al. 2002). In turn, the response and resilience of grasslands to drought are influenced by community composition and diversity (Tilman and Downing 1994; Isbell et al. 2015). Species drought resistance, i.e., their ability to withstand periods of low water availability, varies widely, and species segregate across moisture gradients even within grassland communities (Buckland et al. 1997; Silvertown et al. 2015). The intensity and frequency of drought events are expected to increase with climate change for many regions, including temperate grasslands (IPCC 2014). It remains unclear to what extent the consequences of such extreme drought events can be extrapolated from studies under moderate drought conditions (Williams and Jackson 2007; Slette et al. 2019). There is, thus, an urgent need to improve our understanding of the responses of grassland species to drought.

Performance trade-offs are central in explaining species sorting along environmental gradients and species coexistence (Levins and Culver 1971; Chesson 1985; Rees et al. 2001). A prominent trade-off that has been suggested is a trade-off between growth rates under optimal conditions and tolerance against stress (i.e., fast-growing species are stress intolerant, ‘growth–stress tolerance’ trade-off hypothesis; Grime and Hunt 1975). This trade-off is consistent with the concepts of resource acquisition vs. conservation strategies or the slow vs. fast plant economics spectrum (Craine 2009; Reich 2014). On the other hand, a trade-off between species relative growth rate at different resource levels has been proposed to lead to species rank reversals along gradients of resource availability (‘growth rates’ trade-off hypothesis; Latham 1992). Despite their pervasive implications for competition, community dynamics and species distributions under changing moisture regimes, these trade-offs have rarely been empirically studied concerning drought. Studies are especially missing at the level of whole-plant performance, which is the most directly relevant level for driving ecological patterns and processes (e.g., Kneitel and Chase 2004). For drought, to our knowledge, only two studies have explicitly tested the ‘growth–stress tolerance’ trade-off hypothesis based on growth and survival in grassland species, and both did not find a trade-off (Fernández and Reynolds 2000; Zwicke et al. 2015, in eight and seven species, respectively). The ‘growth rates’ trade-off has been tested in one study in grassland species, which found a strong positive correlation of growth under high and low moisture conditions, rather than a trade-off (Reader et al. 1993). A lack of comparative datasets on species whole-plant drought responses hinders further testing these central hypotheses in plant ecology.

Associations of species’ local- and large-scale distribution patterns with soil moisture are among the most prominent biogeographic patterns (Silvertown et al. 2015 and references therein). Direct effects of moisture on plant performance as well as indirect effects of biotic or other abiotic factors correlated with moisture (e.g., pathogens, nutrients, light; Normand et al. 2009; Silvertown et al. 2015) may lead to these patterns. Linking species whole-plant drought resistance, which reflects species fundamental niche regarding drought, to their distribution across moisture gradients (i.e., their realized niche) allows us to differentiate to what extent differences in species fundamental drought resistance directly determine species distribution across moisture gradients (Engelbrecht et al. 2007a; Esquivel-Muelbert et al. 2017a).

Drought periods can act as a filter, excluding drought-sensitive species from drier sites (‘physiological tolerance hypothesis’, Currie et al. 2004). On the other hand, the occurrence of drought-resistant species under moist conditions may be limited by trade-offs between drought resistance and optimal growth rates, or between performances under different moisture levels (see above). Trade-offs between drought resistance and tolerances to other abiotic (e.g., low nutrients or light) or biotic factors could also exclude drought-resistant species from moist sites (e.g., Silvertown et al. 2015; Grubb 2016; Gaviria et al. 2017). This should lead to a turnover of species with increasingly lower drought resistance along moisture gradients, resulting in a negative relationship between species drought resistance and their association to moist conditions. Evaluation of the direct role of species differential drought resistance for their distributions is still outstanding in grasslands, although it is fundamental to projecting consequences of changes in drought regimes.

In this study, we quantified drought responses of 42 common temperate grassland species by comparing survival (whole-plant and aboveground), and relative growth rate under dry and irrigated conditions in a common garden experiment. This approach allowed us to assess the importance of drought effects on species performance, independent of effects of other abiotic (e.g., light and nutrients) and biotic factors (e.g., plant–plant interactions, herbivores or pathogens, Engelbrecht et al. 2005).

We tested the hypotheses that (1) species differ in their drought responses (i.e., some species are sensitive but others resistant to drought), (2) the ranking of species drought performance remains consistent with increasing drought duration, (3) there are performance trade-offs with respect to drought, specifically (a) a ‘growth–stress tolerance’ trade-off, and (b) a trade-off between growth rates under high and low water availability, and (4) species drought resistance is negatively related to their local- and large-scale association with moisture (i.e., Ellenberg *F* values and rainfall niche).

## Materials and methods

### Field site

The experiment was conducted in a meadow in the Ecological Botanical Garden of the University of Bayreuth, Germany (49°55′19″ N, 11°34′55″ E) in 2015. The area has a temperate climate with 745 mm mean annual precipitation and 8.7 °C mean annual temperature (1998–2007, data: EBG). Rainfall occurs mostly in the growing season with mean monthly rainfall between 60 and 85 mm. Mean monthly temperature ranges between − 0.1 and 17.8 °C with July being the warmest month. In the study area, 32 days or 42 consecutive days without rain during the growing season are considered extreme meteorological drought events based on the 100-year and 1000-year recurrence, respectively (Jentsch et al. 2011)—compared to a 67-day dry treatment in our study (see below). The 2015 summer was extremely hot and dry in Central Europe (Orth et al. 2017), which was reflected in the local conditions during the experiment (Table S1). For conditions in the experiment, see below.

### Study species

We selected 42 perennial species, 19 grasses (Poaceae) and 23 forbs (8 families including five legume species; Table S2), according to the following criteria: (a) common species in German grasslands, based on 150 grassland plots in Germany (Socher et al. 2012), (b) local habitat association with a wide range of moisture conditions (based on Ellenberg indicator values for moisture, *F* values ranging from 3 to 7, Ellenberg et al. 1991), and (c) to include grasses and forbs including legumes and non-legumes. Grasses and forbs did not differ in moisture associations (median *F* value = 5 for both).

Seeds were purchased from commercial seed suppliers (Rieger-Hofmann GmbH and Saaten Zeller, Germany, and Cruydt-Hoeck, Netherlands). They were germinated and grown in the greenhouse for 3 months in the same substrate used in the field experiment (see below). Similar size individuals were selected within each species for the experiment to reduce effects of size variability.

Two species exhibited poor performance already in the greenhouse and had less than 70% survival even under irrigated conditions (Table S3). We, therefore, excluded them from the analyses presented in the text. Analyses with and without them yielded qualitatively the same results (see supplementary material).

### Experimental design

The goal of the experiment was to expose all species to uniformly severe drought conditions to assess whole-plant drought responses in a way that is directly comparable across all species and independent of species interactions. We defined drought as a decrease of water input that leads to a decline of soil moisture, a definition that is commonly used in plant sciences (Gilbert and Medina 2016). Whether the decline of soil moisture affects a plant is determined by its characteristics and can vary between species. We did not aim to mimic a specific natural drought event or climate change regime. By exposing the plants to experimental drought in the field, we avoided common problems associated with drought experiments in pots, namely that soil water depletion is strongly influenced by plant size and differences in transpiration rates, hindering meaningful comparisons among species (Comita and Engelbrecht 2014).

Seedlings were transplanted to 72 plots and exposed to two treatments: a dry treatment, where irrigation was discontinued for 10 weeks in the late summer (36 plots), and an irrigated treatment, where high and favorable water availability was maintained throughout the experiment (36 plots). The plots (1 m × 2 m) were dug out to 1-m depth and filled with sand (97% sand, 2% silt and 1% clay) to ensure that all plants were exposed to uniform soil, and that they dry down to stressfully low levels of water availability (i.e., through low water holding capacity of sand compared to the local loamy soil). All plots were located under transparent rain-out shelters so that both treatments experienced the same light and temperature conditions (see below). Seedlings were planted at 20-cm distance in a rectangular grid (Fig. S1) to minimize plant–plant interactions (i.e., leaves and roots were not overlapping among individuals), thus allowing us to assess the fundamental drought responses of the species.

One individual of each species was planted into each plot (i.e., aiming for 36 individuals per species in each treatment), with species randomly assigned to the grid points. Treatments were blocked to avoid cross-effects of irrigation on dry plots: two plots were set under each shelter, and six shelters were blocked for a treatment (in total six blocks with 36 shelters; see Fig. S1). Plots under each shelter were set up at 0.5-m distance, and shelters and blocks had 1-m and 2-m distance to each other, respectively.

Seedlings were transplanted in the first week of June 2015, and all were regularly irrigated to allow for establishment in the soil. Irrigation was implemented with a drip-irrigation system, with the amount of irrigation adjusted individually for each plot and according to weather conditions to ensure optimal moist conditions, avoiding both superficial soil drying and waterlogging (based on inspection at least five times a week, higher irrigation on warmer/sunnier days). Irrigation was discontinued in the dry treatment plots from 03 Aug 2015 to 09 Oct 2015 (10 weeks). At the end of the drought treatment, we rewatered all plots and removed plastic covers, so that all plots were exposed to natural precipitation until the next spring.

The rain-out shelters (3 m × 3.5 m size, 2.1-m high at the highest point) were covered with transparent plastic foil (200 μm; Gewächshausfolie UV5, folitec Agrarfolienvertriebs GmbH, Westerburg, Germany) with a light transmittance of 86% (assessed with AP4, Delta-T, Cambridge). To allow for air circulation, two sides of the shelters were entirely open, and the others were covered down to 50 cm above the soil. Slow release fertilizer (Terra Plus^®^N; N:P:K 12:4:6%) was applied twice before the start of the treatments (30 g m^−2^) to minimize potential nutrient limitation. Plots were regularly weeded and surrounding areas were mowed to avoid competition from non-target species, and fenced to exclude mammalian herbivores such as deer or hares.

### Environmental conditions in the experiment

We monitored soil water status with gypsum blocks (GB-1 and KS-D1, Delmhorst, NJ), installed at 15-cm soil depth in every plot and additionally at 30-cm depth in six haphazardly chosen plots in each treatment. Readings were taken every 3–5 days around midday and were converted to soil water potentials based on calibration curves. In the irrigated plots soil water potentials remained above − 0.04 MPa throughout the experiment. Dry plots reached soil water potentials below − 1.5 MPa (the permanent wilting point, Veihmeyer and Hendrickson 1928) after 26 ± 9 days (mean ± SD). We additionally characterized midday leaf water potentials in the dry treatment on 3–8 individuals per species in weeks 2–3 of the experimental drought period with leaf cutter psychrometers (Merrill Specialty Equipment, Logan, Utah, USA) and a PSYPRO™ water potential system (Wescor, Inc., Logan, Utah, USA). Midday leaf water potentials varied strongly across species and already reached low values, with species means ranging from − 1.1 to − 5.7 MPa (Sun et al. 2020).

Under the rain-out shelters, daily mean air temperature was 19.4 °C and daily mean relative humidity 74.7% (both assessed with i-buttons, DS1920, Maxim Integrated, CA) with no difference between the treatments (*t* tests, *P* > 0.3 for both).

### Assessments of plant performance and drought resistance

We assessed plant performance based on survival (whole-plant and aboveground) and relative growth rates (RGR). We also classified visually observed drought damage, i.e., leaf wilting and necrosis (modified from Engelbrecht and Kursar 2003, and IRRI 1996, see Table S4). Drought damage and aboveground survival were assessed in all plots once per week during the drought experiment. To differentiate individuals where all aboveground organs died but belowground meristems survived (i.e., aboveground mortality) from individuals where even belowground organs died (i.e., whole-plant mortality), we rechecked all plants for resprouting in the next growing season (June 2016). In the following, the term whole-plant survival refers to individuals that survived the drought (assessed in the next growing season), and aboveground survival refers to individuals that maintained living aboveground biomass during the drought. We quantified species’ survival in the irrigated and dry treatment as the percentage of individuals that survived (whole-plant or aboveground) in the respective treatment relative to the initial number of individuals.

We monitored relative growth rate (RGR) between the first week and sixth week of the treatment (~ 42 days, equivalent to a 1000-year extreme, see above) for all individuals in a subsample of six plots for each treatment (see Table S2 for specific sample sizes). We assessed RGR based on the increase (or loss) of the plants’ projected green leaf area (LA). This non-destructive method allowed for repeated monitoring of growth and survival on the same individuals. LA was determined as the area of an octagon with the focal plant in the center, and with the endpoints of living leaf area along eight plant radii (in 45° angles) representing the corners (compare Breitschwerdt et al. 2018). Tillers were included in the projected LA. We calculated RGR (cm^2^ cm^−2^ day^−1^) from the consecutive measurements in each individual as RGR = (LA_2_ − LA_1_)(LA_1_)^−1^ (*T*_2_ − *T*_1_)^−1^ (Hunt 1978), where LA_1_ and LA_2_ are projected green leaf area at time *T*_1_ and time *T*_2_. Species RGR under irrigated conditions (i.e., combined with high light and nutrients) was significantly correlated with comparative assessments of maximum growth rates (RGR_max_) in a subsample of 24 of the study species in Grime and Hunt (1975; *r* = 0.41, *P* < 0.05), supporting that the non-destructive method usefully captured comparative growth rates. We focused the analyses on RGR of surviving individuals, i.e., the individuals that will contribute to future population dynamics. This parameter does not capture the loss of leaf area (or biomass) occurring in the plants that died, which is relevant for a community or ecosystem perspective. We, thus, additionally analyzed RGR based on all individuals including dead ones (LA = 0). RGR based on survivors and all individuals was highly correlated, and all results with or without including dead individuals were qualitatively similar. All analyses in the text refer to survivors only.

We quantified species comparative drought resistance (DR) as the response ratio of survival (whole-plant, DR_s.whole_ or aboveground, DR_s.above_) or of growth (DR_growth_) in the dry relative to the irrigated treatment (compare Engelbrecht and Kursar 2003). Thus, drought resistance of survival was calculated as DR_s_ = % survival_dry_/% survival_irrigated_. Drought resistance of growth was calculated as DR_growth_ = RGR_dry_/RGR_irrigated_, where RGR_dry_ and RGR_irrigated_ were the median RGR in each treatment because RGR was not normally distributed.

### Species distribution across moisture gradients

To characterize the species association with moisture at the local habitat scale and a large scale, we used Ellenberg indicator values for moisture (*F* value, Ellenberg et al. 1991, see Table S1) and the species rainfall niches, respectively. *F* values are highly correlated with species distributions across directly measured moisture gradients in Europe and can, thus, be considered reliable indicators of species local habitat moisture association (Diekmann 2003). Species that showed no association with soil moisture (*F* value = X) were excluded from the respective analyses. Species rainfall niches were assessed at a spatial resolution of 1 km^2^ based on overlaying annual rainfall from 1979 to 2013 (CHELSA version 1.2, Karger et al. 2017) on species distribution maps (extracted from the GBIF database, using the rgbif package, Chamberlain et al. 2019). The climate data matrix for the focal species was assembled with the raster package (Hijmans 2017). The mean, median, 5th percentile and 95th percentile of the rainfall niche were assessed for each species (Table S1).

### Statistical analyses

Our main aims were to quantify fundamental drought responses of the species and their role in performance trade-offs and species distributions across moisture gradients. We first tested the effects of treatment, species and their interactions (treatment × species) on survival (whole-plant and aboveground), and growth by fitting binomial generalized linear mixed-effects models (GLMM) and linear mixed models (LMM), respectively, using the lme4 package (Bates et al. 2015). Treatment and species were used as fixed effects, and blocks and plots nested in blocks as random effects in each model. The significance of the fixed factors in GLMM was calculated with a Wald test on the full model (Bolker et al. 2009). To additionally assess if treatment and species differences also occurred within life forms, we fitted the models (GLMM and LMM, see above) separately for grasses and forbs. We also assessed the significance of the effect of the drought treatment on survival and growth on each individual species, using separate models for each species with treatment as a fixed and block as a random effect.

We tested if species ranking of drought performance stayed consistent over the duration of the experimental drought. We examined Spearman’s rank correlations between various measures of drought performance after different durations of the dry treatment (weeks 2–9) since data were not normally distributed even after log transformation. We also related them to drought survival (whole-plant and aboveground after 10 weeks) or drought resistance with respect to survival and growth. The measures of drought performance were: % individuals without wilting, % individuals without necrosis, and % aboveground survival (for drought-damage categories see Table S4).

To test the relations between growth under optimal conditions and drought resistance of survival (RGR_irrigated_ vs. DR_s.whole_, ‘growth–stress tolerance’ trade-off hypothesis), and between species’ growth under irrigated and dry conditions (RGR_irrigated_ vs. RGR_dry_, ‘growth rates’ trade-off hypothesis), we used linear regressions. RGR_irrigated_, which was measured under concurrently high moisture, light and nutrient conditions, was used as growth rate under optimal conditions in the test of the ‘growth–stress tolerance’ trade-off. We conducted analyses across species and separately for each life form. We additionally tested the relation of RGR_irrigated_ with visually assessed drought performance after different duration of the dry treatment as alternative measures of drought tolerance (see above).

To test the effects of drought resistance, life form and their interactions (drought resistance x life form) on species moisture association (i.e., *F* value and rainfall niche), linear regression analyses were performed. Ellenberg indicator values can be treated as quasi-metric data in correlations and regressions (Ellenberg et al. 1991; Diekmann 2003; Bartelheimer and Poschlod 2016). We thus analyzed the effects of species drought resistance (DR_s.whole_, DR_s.above_ and DR_growth_), life forms (grass and forb), and their interaction on species moisture association, separately for the *F* values, and the mean, median, 5th percentile, and 95th percentile rainfall niche.

We visually evaluated the normality of the residuals in all analyses and DR_growth_ was consequently log-transformed to improve normality. To avoid negative values, 4 was added to DR_growth_ as a constant value before applying the log transformation.

All statistical analyses were performed in R version 3.3.3 (<https://www.r-project.org/>).

## Results

### Drought damage and consistency of species drought responses over time

**Fig. 1:**
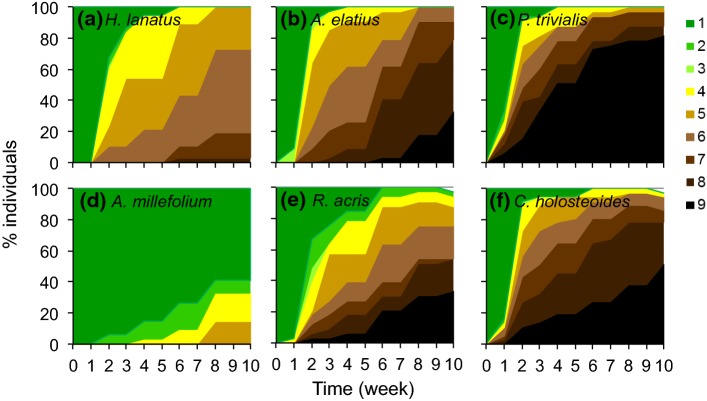
Progression of visual damage in six selected temperate grassland species over 10 weeks of drought, as examples. Shown are the % individuals in different drought damage categories (see Table S4) represented by the color scale, from no visual sign of stress (1, dark green) through progressive signs of wilting or rolling, and tissue necrosis to complete death of all aboveground biomass (9, black). Grasses are presented in the first row and forbs in the second row. For species codes, see Table S1. Shown are two examples for species with low aboveground mortality and either **a** early visual signs of drought damage or **d** few and late signs of stress, two examples for intermediate species with **b**, **e** moderate aboveground mortality and early drought damage, and two examples of species with **c**, **f** high aboveground mortality and early drought damage. See Fig. S2 for graphs of all species. Color version of this figure is available online

All species exhibited wilting and necrosis in the dry treatment. Its extent and the progression varied strongly among species (Fig. 1, Fig. S2). While some species exhibited pronounced wilting and extensive tissue necrosis with complete aboveground mortality in many individuals, others showed only little wilting and slight necrosis (Fig. 1, Fig. S2).

Survival throughout the intense dry treatment was overall high, both for the whole plants (see below for details) and for aboveground organs. More than half of the species had higher than 90% aboveground survival. Of those species where some individuals exhibited complete aboveground mortality, 83% exhibited resprouting (Table S2, Fig. S3).

**Fig. 2:**
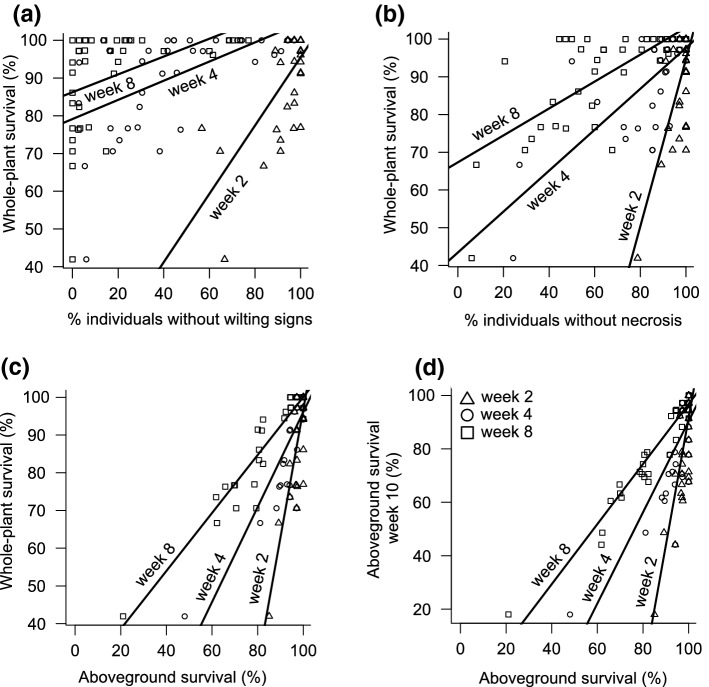
Examples for **a**–**c** relations between whole-plant drought survival and performance after different durations of drought, and **d** between aboveground survival after 10 weeks and aboveground survival after shorter drought periods. Shown are relations for **a** % individuals without any signs of wilting, **b** % individuals without necrosis and **c**, **d** % aboveground survival. Reponses are indicated for three of the drought durations. All Spearman’s rank correlations were significant (*P* < 0.01). See Table S5 for the complete correlation tables. For visualization, regression lines are shown

Species ranking of drought performance stayed consistent across different durations of the dry treatment (see Fig. 2 for examples). Aboveground survival at different time points into the drought was highly positively correlated with each other across species, as well as with whole-plant survival and with drought resistance for survival (*r* > 0.8, *P* < 0.001 for all, Table S5). Similarly, the percentage of individuals without wilting or necrosis at different time points into the drought was highly positively related to each other across species, and to the final survival (whole-plant and aboveground) at the end of the drought (Table S5). On the other hand, drought damage and aboveground survival were consistently unrelated to drought resistance for growth (Table S5).

### Drought effects on plant survival and growth

**Fig. 3:**
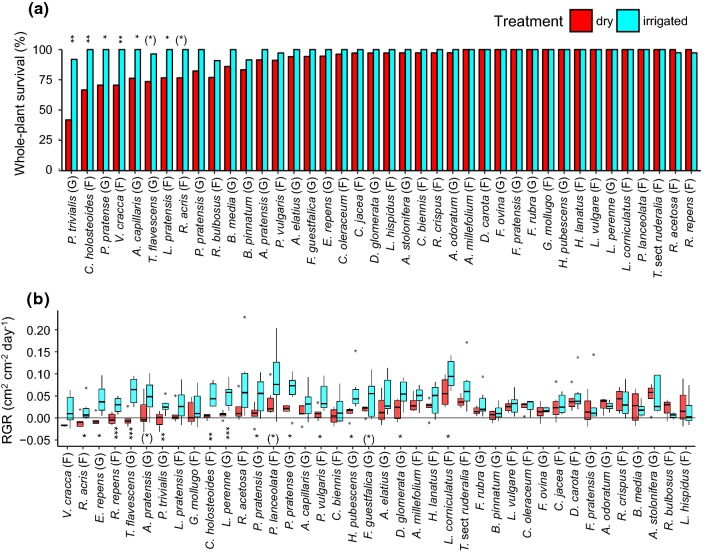
**a** Survival and **b** relative growth rate (RGR, median, and the 25% and 75% quartiles) of 40 perennial temperate grassland species under dry and irrigated conditions. Given are % individuals that survived (at the whole plant level) relative to the initial number of individuals, and RGR based on projected living leaf area. Species are ordered according to their drought resistance (compare Fig. S4), i.e., from large to small responses to the dry relative to the irrigated treatment, for survival and RGR, respectively. Grasses and forbs are indicated with G and F, respectively. Significance of treatment effects within each species are given as (*)*P* < 0.1, *P < 0.05, **P < 0.01. For details see Table S7. For overall treatment and species effects see Table 1. Color version of this figure is available online

Even after the intense dry treatment, 70% of the species exhibited more than 90% whole-plant survival (Fig. 3a). RGR was positive in most species even under dry conditions (to week 6). Net losses of projected leaf area occurred only in five species (12%). Nevertheless, across all species, the dry treatment had a significant negative effect on survival (whole-plant and aboveground) and growth (Table 1). Species significantly differed in survival and growth without significant treatment x species interactions (Table 1, Table S6). Similar results emerged in grasses separately, but in forbs, the drought had no significant effect on whole-plant survival and RGR.

**Table 1:** Effects of drought treatment, species, and their interaction on survival (whole-plant and aboveground) and relative growth rate in 40 grassland species

	Treatment	Species	Treatment × species
All species			
Whole-plant survival	20.44**	122.91***	11.81
Aboveground survival	38.05***	217.27***	25.23
Relative growth rate	7.52**	2.75***	1.09
Grasses			
Whole-plant survival	16.20**	74.71***	9.37
Aboveground survival	27.72***	124.86***	19.75
Relative growth rate	11.27**	1.22	1.20
Forbs			
Whole-plant survival	3.54	34.92*	1.50
Aboveground survival	20.68**	88.54***	5.82
Relative growth rate	4.49	4.09***	1.02

Regression models (GLMM and LMM) were run across all species and additionally for the two life forms (grasses and forbs), separately. Blocks and plots nested in blocks were included as random effects in each model. Given are Chi^2^ and *F* values for survival and growth, respectively. Significance levels are indicated as *P < 0.05, **P < 0.01, ***P < 0.001. See Table S6 for analyses over all initial 42 species

Within individual species, the dry treatment had a significant negative effect on whole-plant survival and aboveground survival in 15% and 30% of the species, respectively (Fig. 3a, Table S7). RGR was significantly negatively affected by drought in 34% of the species (Fig. 3b, Table S7).

Drought resistance varied continuously across species for whole-plant survival (DR_s.whole_), aboveground survival (DR_s.above_), and growth (DR_growth_; Fig. S4). All parameters did not vary between life forms (*t* tests, *P* > 0.1).

### Testing for performance trade-offs with respect to drought

**Fig. 4:**
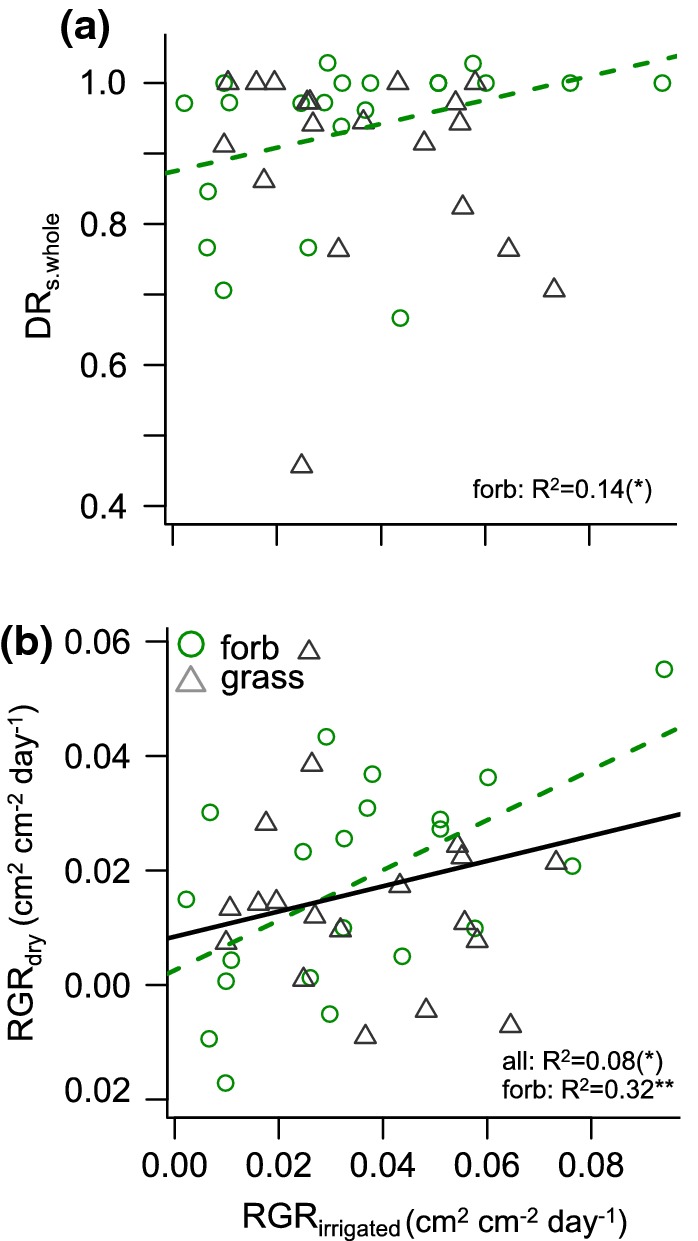
Tests for performance trade-offs across 40 perennial temperate grassland species. Relations are shown **a** between growth under irrigated conditions and drought resistance of survival (RGR_irrigated_ vs. DR_s.whole_; test of ‘growth–stress tolerance’ trade-off hypothesis), and **b** between growth under irrigated and under dry conditions (RGR_irrigated_ vs. RGR_dry_; test of ‘growth rates’ trade-off hypothesis). RGR is given as cm^2^ cm^−2^ day^−1^ based on projected living leaf area. Significant relations across all species are given as black solid lines, within forbs as green dashed lines (no significant relations for grasses). *R*^2^ and significance levels are given as (*)*P* < 0.1, **P < 0.01. For relations of RGR_irrigated_ to additional measures of drought tolerance see Table S5

We found no indication of a trade-off between optimal growth and drought survival. Growth in the irrigated treatment (RGR_irrigated_) and drought resistance for whole-plant survival (DR_s.whole_) were unrelated across all species and within grasses, and even marginally positively related within forbs (Fig. 4a, Table S8). DR_s.whole_ was also unrelated to RGR_max_ from the literature (*P* > 0.1, 24 species from Grime and Hunt 1975), further supporting our results. Similarly, there was no trade-off between RGR_irrigated_ and other measures of species drought tolerance, i.e., % individuals without wilting, % individuals without necrosis or % aboveground survival (Table S5).

Species growth rates in the irrigated and the dry treatment (RGR_irrigated_ vs. RGR_dry_) were marginally significantly positively related across species (Fig. 4b), indicating no trade-off between growth rates under high and low moisture conditions. Within forbs, the relation was even significantly positively; while no relation emerged within grasses.

### Testing for relations of drought resistance to species moisture association

**Fig. 5:**
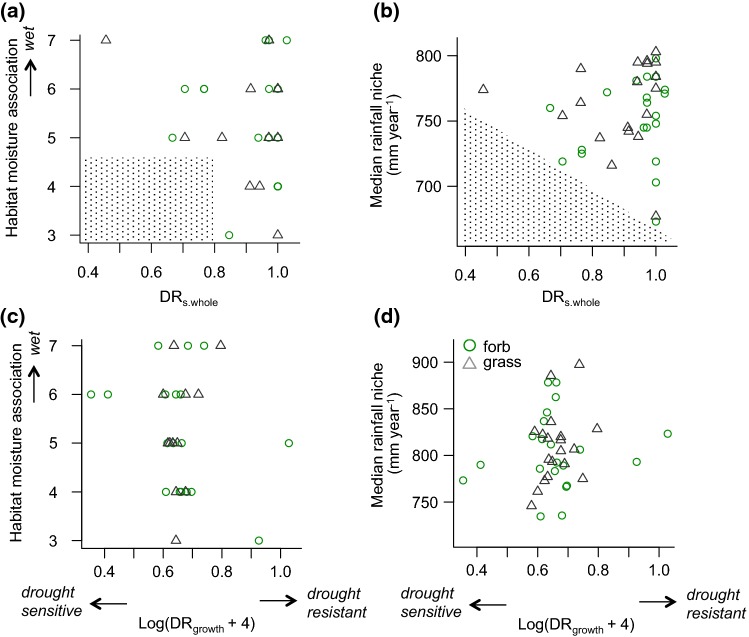
Relations of species drought resistance with their distribution across moisture gradients. Results are given for **a**, **b** drought resistance with respect to whole-plant survival (DR_s.whole_), and **c**, **d** with respect to growth (DR_growth_). Distribution across moisture gradients is characterized at the local level based on **a**, **c** the Ellenberg *F* value (with larger values indicating moister conditions) and **b**, **d** at the large scale based on their median annual rainfall niche. None of the relations was significant (all *P* >> 0.1). The stippled areas in **a** and **b** illustrate that no drought-sensitive species were associated with dry habitats or exhibited low rainfall niches (see Fig. S5 for relations to 5th percentile, mean and 95th percentile rainfall niches)

Drought resistance with respect to whole-plant survival, aboveground survival or growth (DR_s.whole_, DR_s.above_, DR_growth_) was not related (*P* > 0.1) to species local habitat moisture association (*F* value; Fig. 5a, b) nor to the large-scale rainfall niche (Fig. 5c, d for the median, Fig. S5 for the mean, 5th, and 95th percentile). Life form had no significant effect on species local- or regional-scale moisture association, and there was no drought resistance × life form interaction (all *P*  >> 0.1).

## Discussion

### High drought resistance of temperate grassland species

Drought had an overall negative impact on survival (both whole-plant and aboveground) and growth of common temperate grassland species. Wilting and leaf necrosis increased with drought duration. However, high survival and positive growth under the drought in most species indicate that many species in temperate grasslands are well adapted to even intense drought conditions. These findings are consistent with previous studies, which reported reduced biomass productivity during drought, but high recovery after the drought for natural and experimental temperate grassland communities (Grime et al. 2008; Kreyling et al. 2008; Gilgen and Buchmann 2009; Vogel et al. 2012; Hofer et al. 2016). Nevertheless, individual species responded differentially to drought in terms of survival and growth. Differential responses of species to drought have been suggested to alter species distribution and the composition and diversity of grassland communities (Silvertown et al. 1999; Grime et al. 2000; Hoover et al. 2014).

To our knowledge, this is the most extensive study that directly and experimentally assessed comparative whole-plant responses of individual species in grasslands. It allowed for the first time to rigorously test trade-offs between performance parameters concerning drought, and the relation between species drought resistance and distributions across moisture gradients in temperate grassland species.

The performance of the species in our drought experiment does not necessarily reflect their performance under natural extreme drought events of comparable length, because the soil water status and the soil–atmosphere water potential gradients that plants experience are highly dependent on actual weather conditions, soil characteristics and site hydrology. Nevertheless, the ranking of species drought damage and aboveground survival remained consistent across a wide range of drought durations, from those frequently occurring in the study area to ones exceeding 1000-year events (Fig. 2, Table S5). Thus, studies that assess comparative drought survival of perennial grassland species under moderate conditions can be used to infer drought survival of individual species under more intense conditions even beyond those experienced under current climate conditions (compare Slette et al. 2019). The results imply that early leaf abscission is not a dominant survival strategy in temperate grassland species, which contrasts with Mediterranean grassland species (Volaire et al. 2009). They also suggest that previous studies, which evaluated the importance of traits for aboveground survival, are also relevant at the whole-plant level (e.g., Belluau and Shipley 2017).

Furthermore, our results show that visually assessed drought damage (i.e., wilting and necrosis) after short drought periods (i.e., a few weeks) is a useful proxy for plant survival of longer drought in temperate grassland species, consistent with results in tropical seedlings (Engelbrecht et al. 2007b). The proxy can facilitate studying effects of extreme droughts on grassland species, e.g., for selecting species to improve grassland resilience to drought, for further testing how species fundamental drought resistance translates into species abundance and distribution patterns, or for evaluating the importance of traits for drought survival. On the other hand, the ranking of species responses of relative growth rates was not related to those of visual damage or aboveground survival (Table S5). Competitive interactions may, therefore, change with drought duration or intensity, and impede inferring community responses to extreme drought events from studies under moderate drought conditions.

### No performance trade-offs with respect to drought

We found no indication for a trade-off between relative growth rates in the irrigated treatment and drought resistance (‘growth–stress tolerance’ trade-off, Fig. 4a, Table S5) nor for a trade-off between relative growth rates in the irrigated and dry treatment (‘growth rates’ trade-off, Fig. 4b). This was despite a large (> fivefold) variation in growth rates of well-watered plants across species, as well as extreme drought conditions. Some trends were even in the opposite direction to the hypotheses.

Although the lack of trade-offs was unexpected, it was indeed consistent with previous studies at the whole-plant level in relatively small species sets (Fernández and Reynolds 2000; Zwicke et al. 2015). Indirect evidence for the ‘growth–stress tolerance’ trade-off had been previously been provided by a positive relationship between species moisture association with relative growth rates in temperate grassland species (Bartelheimer and Poschlod 2016) and tropical woody seedlings (Gaviria et al. 2017). At the trait level, evidence for a trade-off between some traits enabling fast growth and proxies of drought resistance was also reported for grassland species (Craine et al. 2013), C_4_ grasses (Ocheltree et al. 2016), and woody species (Reich 2014 and references therein).

Plants cope with drought through various different processes such as minimizing water loss, maximizing water uptake and transport, and maintaining carbon gain (Choat et al. 2018). Morphological, anatomical, and physiological traits that are relevant for these processes not only can be positively correlated among each other or trade-off, but also can vary independently (Tucker et al. 2011; Zwicke et al. 2015). Also, many relevant traits exhibit phenotypic plasticity in response to varying soil moisture (Jung et al. 2014; de Vries et al. 2016). The various processes involved in growth and drought resistance, as well as the complexities in trait relations and plasticity, may lead to the independent variation of species drought resistance and maximum growth rate at the whole-plant level.

A ‘growth rates’ trade-off indicated by performance rank reversals across resource gradients has been hypothesized and should lead to shifts of competitive hierarchies (Latham 1992). In contrast, we found that species growth rates in the dry and irrigated treatments were independent of each other in grasses, and were even positively related in forbs and across all species. Studies testing the ‘growth rates’ trade-off hypothesis in grassland species found a rank reversal under nutrient-rich vs. nutrient-poor conditions, but not under different water and light conditions (Reader et al. 1993; Meziane and Shipley 1999). Studies on woody species concerning drought or shade (Dalling et al. 2004; Baraloto et al. 2006; Kitajima and Poorter 2008) and on herbaceous wetland species regarding nutrients (Keddy et al. 2000) also found no evidence of consistent rank reversals of growth rates across resource levels.

The lack of a ‘growth–stress tolerance’ or ‘growth rates’ trade-off with respect to drought implies that these performance trade-offs cannot be the dominant drivers of hydrological niche differentiation or maintenance of diversity in temperate grasslands, and that instead other factors are more important (compare Silvertown et al. 2015). These may include nutrients or pest pressure, as well as temporal fluctuations in limiting factors (i.e., storage effect), competition–defense trade-offs or negative density dependence (Harpole and Tilman 2007; Chesson and Kuang 2008; Adler et al. 2013).

### No relations of drought resistance to species moisture association

Species drought resistance is expected to shape their distribution across moisture gradients (e.g., Silvertown et al. 1999; Hoover et al. 2014; Esquivel-Muelbert et al. 2017a). Relations of some physiological traits that are related to water relations and gas exchange with species’ habitat moisture support this expectation (e.g., stomatal behavior; Tucker et al. 2011; Belluau and Shipley 2017; but see Májeková et al. 2019). Nevertheless, we found no indication that species drought resistance was negatively related to moisture associations at the local or large scale (Fig. 5, Fig. S5).

The distribution of species with high drought resistance in terms of survival (DR_s.whole_) reached into rather moist areas both at the local and large scales (Fig. 5, Fig S5). This implies that drought-resistant grassland species are not systematically excluded from moist areas by trade-offs against drought resistance (see the introduction, Gaviria et al. 2017), a result consistent with the observed lack of ‘growth–drought tolerance’ or ‘growth rates’ trade-offs (see above).

On the dry side, no drought-sensitive species (DR_s.whole_ < 0.8) were associated with dry habitats (*F* values 3 and 4, Fig. 5a) or exhibited low rainfall niches (median < 700 mm year^−1^, 5th percentile < 550 mm year^−1^, Fig. 5b, Fig. S5a). Although this pattern was weak, it is consistent with drought directly excluding drought-sensitive species from dry habitats. Overall, however, our data suggest that in temperate grassland species differences of their fundamental drought resistance are not a main driver of their distribution across moisture gradients.

These results on temperate grassland species differ from findings from moist tropical forests, where species drought resistance had a pronounced direct effect on their distribution along regional- and large-scale rainfall gradients (Engelbrecht et al. 2007a; Esquivel-Muelbert et al. 2017a), underlining that the relative importance of mechanisms for species distributions differs across ecosystems. However, consistent with our results, drought-resistant species were also not excluded from wet areas, indicating that any potential trade-offs of drought resistance with other factors (e.g., growth rates or shade tolerance) were similarly weak (Esquivel-Muelbert et al. 2017b).

Besides the direct interplay between plant drought resistance and moisture, various additional factors that co-vary with water availability can limit the distribution of individual species along moisture gradients. Complex interactions of drought resistance with other resource requirements or tolerances can also accelerate or dampen effects of water availability (e.g., Eskelinen and Harrison 2015). For example, high nitrogen requirements may limit the distribution of a drought-resistant species to water regimes that are suitable for high microbial nitrogen mineralization rates and at the same time allow high nitrogen uptake with the transpiration stream (Araya et al. 2013). Additionally, plant–plant interactions, i.e., competition and facilitation, are known to play an important role in the performance of grassland species across moisture gradients and to influence species distributions (e.g., Brooker et al. 2008). Thus, multiple processes operate simultaneously to structure species realized distribution and plant communities along this resource gradient (Spasojevic and Suding 2012).

Nevertheless, existing relations between species drought resistance and moisture associations may have been obscured in our data because both available measures of species moisture associations are coarse. The local-scale classification of habitat association is non-quantitative (Ellenberg et al. 1991). The large-scale rainfall niche, although quantitative, has a low spatial resolution (1 km^2^) and thus does not allow resolving the often substantial small-scale variation of soil moisture (e.g., with topography or soil). Additionally, both measures do not refer to soil water potentials, the parameter ultimately relevant for plant water relations (Lambers et al. 2007). To further advance our understanding of the importance of species fundamental drought resistance for their realized distribution across moisture gradients in grasslands, and to differentiate it from other factors, we ideally need species quantitatively assessed abundance changes in response to the spatial and temporal variation of soil water potentials. However, such data are rarely available (but see Kupers et al. 2019).

Experimentally assessed comparative whole-plant drought resistance, as we present in this study for temperate grassland species, provides a basis to further examine the processes that shape community composition and species distributions under different moisture regimes. Especially combining the drought resistance of individual species with their responses to drought in community-level experiments or in natural communities (e.g., Tilman and El Haddi 1992; Bütof et al. 2012; Isbell et al. 2015; Herz et al. 2017) will help to elucidate the various interacting factors. Comparative assessments are also a prerequisite for rigorously testing the importance of traits and trait combinations for whole-plant drought resistance (Shipley et al. 2016). They will, therefore, contribute to improving projections of the consequences of changing moisture regimes for grasslands under climate change.

## Electronic supplementary material

Below is the link to the electronic supplementary material.

Supplementary material 1 (DOC 2613 kb)
