# Supplementary material for: Comparative drought resistance of temperate grassland species: testing performance trade-offs and the relation to distribution
Source: Oecologia. 2020 Feb 29;192(4):1023–36. doi: 10.1007/s00442-020-04625-9 (PMC7165153; doi:10.1007/s00442-020-04625-9)
Supplement: Supplementary file 1 — Supplementary material 1 (DOC 2613 kb) [file 442_2020_4625_MOESM1_ESM.doc]

**Supplemental Materials**

**Comparative drought resistance of temperate grassland species: testing performance trade-offs and the relation to distribution**

Eun-Young Jung, Julian Gaviria, Shanwen Sun, Bettina M. J. Engelbrecht

**Contact information**Eun-Young Jung

Address: Department of Plant Ecology, Bayreuth Center of Ecology and Environmental Research (BayCEER), University of Bayreuth, Bayreuth, 95440, Germany

Email address: [eun-young.jung@uni-bayreuth.de](mailto:eun-young.jung@uni-bayreuth.de)

Telephone: +49-921-55-2581

**Table S1** Comparison of monthly rainfall and average temperature during the experimental period (3-Aug-2015−9-Oct-2015) with the respective long-term averages (1981−2010). Data are from the Ecological Botanical Garden, University of Bayreuth.

|  | **Rainfall (mm)** | | **Temperature (°C)** | |
| --- | --- | --- | --- | --- |
|  | 2015 | Long-term average | 2015 | Long-term average |
| July | 32 | 92 | 19.9 | 17.6 |
| August | 40 | 64 | 20.5 | 16.6 |
| September | 25 | 64 | 12.7 | 12.6 |

**Table S2** List of the study species. Given are the species name, code, family, life form (grasses and forbs, including legumes, L), the Ellenberg’s indicator value for moisture (F-value, Ellenberg *et al*. 1991, ‘X’ indicates ‘indifferent’), and annual rainfall niche (mm year^-1^). The last two species (*M. lupulina, T. repens*) were excluded throughout all analyses presented in the main text because of their poor performance in the greenhouse and irrigated plots.

| **Species** | **Code** | **Family** | **Life form** | **F-value** | **Rainfall niche** | | |
| --- | --- | --- | --- | --- | --- | --- | --- |
|  |  |  |  |  | **Median** | **5^th^ percentile** | **95^th^ percentile** |
| *Achillea millefolium* L. | ACHIMI | Asteraceae | Forb | 4 | 719 | 505 | 1387 |
| *Agrostis capillaris* L. | AGROCA | Poaceae | Grass | x | 764 | 522 | 1515 |
| *Agrostis stolonifera* L. | AGROST | Poaceae | Grass | 7 | 796 | 551 | 1352 |
| *Alopecurus pratensis* L. | ALOPPR | Poaceae | Grass | 6 | 742 | 534 | 1151 |
| *Anthoxanthum odoratum* L. | ANTHOD | Poaceae | Grass | x | 794 | 546 | 1779 |
| *Arrhenatherum elatius* (L.) J. & K. | ARRHEL | Poaceae | Grass | x | 780 | 554 | 1335 |
| *Brachypodium pinnatum* (L.) PB. | BRACPI | Poaceae | Grass | 4 | 745 | 548 | 1302 |
| *Briza media* L. | BRIZME | Poaceae | Grass | x | 716 | 513 | 1309 |
| *Centaurea jacea* L. | CENTJA | Asteraceae | Forb | x | 745 | 533 | 1129 |
| *Cerastium holosteoides* Fr. | CERAHO | Caryophyllaceae | Forb | 5 | 760 | 546 | 1327 |
| *Cirsium oleraceum* (L.) Scop. | CIRSOL | Asteraceae | Forb | 7 | 745 | 555 | 1248 |
| *Crepis biennis* L. | CREPBI | Asteraceae | Forb | 6 | 784 | 558 | 1200 |
| *Dactylis glomerata* L. ssp. *glomerata* | DACTGL | Poaceae | Grass | 5 | 755 | 500 | 1370 |
| *Daucus carota* L. ssp. *carota* | DAUCCA | Apiaceae | Forb | 4 | 754 | 477 | 1184 |
| *Elymus repens* (L.) Gould | ELYMRE | Poaceae | Grass | x | 738 | 523 | 1076 |
| *Festuca guestfalica* Boenn. Ex Rchb. | FESTGU | Poaceae | Grass | 4 | 795 | 562 | 1278 |
| *Festuca ovina* L. agg. | FESTOV | Poaceae | Grass | x | 677 | 462 | 1564 |
| *Festuca pratensis* Huds*.* ssp. *pratensis* | FESTPR | Poaceae | Grass | 6 | 795 | 561 | 1144 |
| *Festuca rubra* L. | FESTRU | Poaceae | Grass | 6 | 775 | 528 | 1446 |
| *Galium mollugo* L. agg. | GALIMO | Rubiaceae | Forb | 4 | 703 | 532 | 1052 |
| *Helictotrichon pubescens* (Huds.) Pilg. ssp. *pubescens* | HELIPU | Poaceae | Grass | 3 | 803 | 543 | 1634 |
| *Holcus lanatus* L. | HOLCLA | Poaceae | Grass | 6 | 798 | 588 | 1518 |
| *Lathyrus pratensis* L. | LATHPR | Fabaceae | Forb (L) | 6 | 725 | 526 | 1330 |
| *Leontodon hispidus* L. | LEONHI | Asteraceae | Forb | 5 | 768 | 556 | 1327 |
| *Leucanthemum vulgare* Lam. | LEUCVU | Asteraceae | Forb | 4 | 673 | 518 | 1161 |
| *Lolium perenne* L. | LOLIPE | Poaceae | Grass | 5 | 784 | 561 | 1334 |
| *Lotus corniculatus* L. | LOTUCO | Fabaceae | Forb (L) | 4 | 784 | 530 | 1678 |
| *Phleum pratense* L. | PHLEPR | Poaceae | Grass | 5 | 754 | 533 | 1219 |
| *Plantago lanceolata* L. | PLANLA | Plantagina-ceae | Forb | x | 784 | 526 | 1552 |
| *Poa pratensis* L. | POAPR | Poaceae | Grass | 5 | 737 | 520 | 1266 |
| *Poa trivialis* L. ssp. *trivialis* | POATR | Poaceae | Grass | 7 | 774 | 547 | 1242 |
| *Prunella vulgaris* L. | PRUNVU | Lamiaceae | Forb | 5 | 781 | 544 | 1707 |
| *Ranunculus acris* L. | RANUAC | Ranuncula-ceae | Forb | 6 | 728 | 519 | 1403 |
| *Ranunculus bulbosus* L. ssp. *bulbosus* | RANUBU | Ranunculaceae | Forb | 3 | 772 | 540 | 1203 |
| *Ranunculus repens* L. | RANURE | Ranunculaceae | Forb | 7 | 774 | 540 | 1445 |
| *Rumex acetosa* L. | RUMEAC | Polygonaceae | Forb | x | 771 | 522 | 1588 |
| *Rumex crispus* L. | RUMECR | Polygonaceae | Forb | 7 | 764 | 543 | 1357 |
| *Taraxacum* sect. *Ruderalia* Kirschner H.Øllg. & Štěpánek | TARARU | Asteraceae | Forb | 5 | 748 | 514 | 1359 |
| *Trisetum flavescens* (L.) PB. | TRISFL | Poaceae | Grass | x | 790 | 572 | 1240 |
| *Vicia cracca* L. | VICICR | Fabaceae | Forb (L) | 6 | 719 | 512 | 1318 |
| *Medicago lupulina* L. | MEDILU | Fabaceae | Forb (L) | 4 | 759 | 532 | 1138 |
| *Trifolium repens* L. | TRIFRE | Fabaceae | Forb (L) | 5 | 762 | 516 | 1501 |

**Table S3 Detailed data on the drought experiment for each species. Given are the number of living individuals (whole-plant level) at the initial and final census in the irrigated and dry treatments, respectively, the number of individuals without living aboveground biomass at the end of the dry treatment, the number of those individuals that resprouted in the next growing season, and the sample sizes for relative growth rate (in parenthesis the number of individuals that died during the dry treatment). In species where the initial sample number for survival was below 36, the full number of seedlings was not available for transplanting, for species with initial sample numbers above 36 a transplanting error had occurred. The last two species (MEDILU, TRIFRE) were excluded throughout all analyses presented in the main text because of their poor performance in the greenhouse and irrigated plots. See Table S6 and S8 for analyses including these species.**

| **Species code** | **Irrigated treatment** | | **Dry treatment** | | **Recovery** | | **RGR** | |
| --- | --- | --- | --- | --- | --- | --- | --- | --- |
|  | **#**  **Initial** | **#**  **Final** | **#**  **Initial** | **#**  **Final** | **w/o living aboveground biomass** | **#**  **Resprouting** | **#**  **Irrigated** | **#**  **Dry** |
| ACHIMI | 37 | 37 | 34 | 34 | 0 | 0 | 6 | 5 |
| AGROCA | 37 | 37 | 37 | 30 | 13 | 6 | 6 | 4 (2) |
| AGROST | 35 | 35 | 36 | 35 | 2 | 1 | 5 | 6 |
| ALOPPR | 33 | 33 | 35 | 32 | 9 | 6 | 6 | 6 |
| ANTHOD | 36 | 36 | 37 | 36 | 2 | 1 | 6 | 5 (1) |
| ARRHEL | 36 | 36 | 34 | 32 | 11 | 9 | 6 | 6 |
| BRACPI | 35 | 32 | 36 | 30 | 8 | 2 | 6 | 6 |
| BRIZME | 36 | 36 | 35 | 31 | 10 | 6 | 6 | 6 |
| CENTJA | 32 | 32 | 36 | 34 | 2 | 0 | 6 | 6 |
| CERAHO | 37 | 37 | 36 | 24 | 20 | 8 | 6 | 4 (2) |
| CIRSOL | 13 | 13 | 26 | 25 | 1 | 0 | 3 | 3 |
| CREPBI | 36 | 36 | 36 | 35 | 2 | 1 | 6 | 6 |
| DACTGL | 36 | 35 | 36 | 35 | 2 | 1 | 6 | 6 |
| DAUCCA | 39 | 39 | 35 | 35 | 1 | 1 | 6 | 6 |
| ELYMRE | 35 | 35 | 36 | 34 | 8 | 6 | 6 | 5 (1) |
| FESTGU | 35 | 35 | 35 | 33 | 3 | 1 | 5 | 6 |
| FESTOV | 37 | 37 | 35 | 35 | 0 | 0 | 6 | 6 |
| FESTPR | 35 | 34 | 35 | 33 | 2 | 0 | 6 | 5 (1) |
| FESTRU | 36 | 36 | 35 | 35 | 2 | 2 | 6 | 6 |
| GALIMO | 34 | 34 | 35 | 35 | 1 | 1 | 6 | 6 |
| HELIPU | 36 | 36 | 34 | 34 | 1 | 1 | 6 | 5 (1) |
| HOLCLA | 37 | 37 | 38 | 37 | 1 | 0 | 6 | 6 |
| LATHPR | 34 | 34 | 31 | 24 | 11 | 4 | 4 | 5 |
| LEONHI | 33 | 33 | 35 | 34 | 1 | 0 | 5 | 6 |
| LEUCVU | 36 | 36 | 37 | 37 | 0 | 0 | 6 | 6 |
| LOLIPE | 36 | 36 | 36 | 36 | 1 | 1 | 6 | 6 |
| LOTUCO | 36 | 36 | 35 | 35 | 1 | 1 | 6 | 6 |
| PHLEPR | 36 | 36 | 35 | 28 | 10 | 3 | 6 | 4 (2) |
| PLANLA | 36 | 36 | 36 | 36 | 0 | 0 | 6 | 6 |
| POAPR | 35 | 35 | 33 | 26 | 10 | 3 | 6 | 5 |
| POATR | 37 | 34 | 31 | 13 | 25 | 7 | 6 | 3 (3) |
| PRUNVU | 35 | 34 | 31 | 29 | 6 | 4 | 6 | 5 (1) |
| RANUAC | 29 | 28 | 30 | 23 | 10 | 3 | 6 | 5 |
| RANUBU | 12 | 11 | 13 | 10 | 3 | 0 | 3 | 3 |
| RANURE | 36 | 35 | 36 | 36 | 6 | 6 | 6 | 6 |
| RUMEAC | 37 | 36 | 36 | 36 | 1 | 1 | 6 | 5 (1) |
| RUMECR | 35 | 35 | 36 | 35 | 1 | 0 | 6 | 6 |
| TARARU | 36 | 36 | 36 | 36 | 4 | 4 | 6 | 5 |
| TRISFL | 34 | 33 | 35 | 25 | 20 | 10 | 6 | 4 (1) |
| VICICR | 37 | 37 | 35 | 25 | 13 | 3 | 6 | 2 (4) |
| MEDILU | 35 | 24 | 30 | 16 | 14 | 0 | 4 | 5 (1) |
| TRIFRE | 33 | 22 | 32 | 4 | 30 | 2 | 5 | 1 (5) |

**Figure S1** Experimental design. A dry and an irrigated treatment were applied to 72 plots, with one individual of each of 42 species randomly assigned to positions in a 20cm x 20cm grid. Plots were located in 6 blocks, 3 blocks for the wet treatment (blue) and 3 blocks for the dry treatment (red). Within each block were six transparent rain-out shelters, and two plots were located beneath each shelter, i.e., 6 blocks x (6 shelters x 2 plots) => 72 plots in total.

**Table S4** Drought-damage categories used in visual assessments. The categories were modified from systems for rice (IRRI 1996) and for tropical tree seedlings (Engelbrecht and Kursar 2003). Columns A, B and C indicate categories included in the analyses as individuals without wilting, individuals without necrosis, and individuals with aboveground survival, respectively (Fig. 2, Table S5).

| **Category** | **A** | **B** | **C** | **Visual characteristics** |
| --- | --- | --- | --- | --- |
| 1 | X | X | X | no signs of drought stress |
| 2 |  | X | X | slight signs of wilting (leaf angle changes, rolling, or folding) without leaf necrosis |
| 3 |  | X | X | strong signs of wilting (leaf angle changes, rolling, or folding) without leaf necrosis |
| 4 |  |  | X | slight leaf tip drying (necrosis) |
| 5 |  |  | X | tip drying (necrosis) extended to ¼ length in up to 25% of the leaves (normally old leaves) |
| 6 |  |  | X | tip drying (necrosis) extended to ¼ length in up to 50% of the leaves (or 25% of leaves fully dried) |
| 7 |  |  | X | more than 50% of the leaves fully dried |
| 8 |  |  | X | more than 70% of the leaves fully dried |
| 9 |  |  |  | all above-ground parts dead |

**Figure S2** Progression of visual drought damage over the 10-week experimental drought in 40 temperate grassland species (expanded from Fig. 1). Shown are the % individuals in different drought damage categories (see Table S4), represented by the color scale, from no visual sign of stress (1, dark green) through progressive signs of wilting or rolling, and tissue necrosis to complete death of all aboveground biomass (9, black). Species are sorted alphabetically, and separately for (a) grasses and (b) forbs.

**Figure S3** Percent resprouting in 16 of the study species, where ≥ 6 individuals exhibited complete aboveground mortality after the extreme drought (compare Table S3).

**Table S5** (a) Correlations among performance measures after progressing duration of the dry treatment, and (b) correlations of the performance measures with aboveground survival after 10 weeks (S_above10_), whole-plant survival (S_whole10_), and with drought resistance with respect to whole-plant survival (DR_s.whole_), aboveground survival (DR_s.above_) and growth (DR_growth_), and with growth rate under irrigated conditions (RGR_irrigated_) across 40 species. Correlation tables are given for (1)% individuals without wilting signs, (2)% individuals without necrosis, and (3) % individuals with aboveground survival. Durations of the dry treatment are indicated in weeks (W2, 3, etc.). Given are Spearman’s rank correlation coefficients (ρ) and significance levels (****P* < 0.001, ***P* < 0.01, **P* < 0.05. (*)*P* < 0.1). Significant relations after Bonferroni correction within each section are in bold. All results were qualitatively the same when considering all initial 42 species (data not shown).

**(1) % individuals without wilting signs**

|  | W2 | W3 | W4 | W5 | W6 | W7 | W8 | W9 |
| --- | --- | --- | --- | --- | --- | --- | --- | --- |
| (a) |  |  |  |  |  |  |  |  |
| W3 | **0.55***** |  |  |  |  |  |  |  |
| W4 | 0.46** | **0.97***** |  |  |  |  |  |  |
| W5 | 0.45** | **0.90***** | **0.93***** |  |  |  |  |  |
| W6 | 0.45** | **0.90***** | **0.93***** | **0.99***** |  |  |  |  |
| W7 | 0.43** | **0.81***** | **0.83***** | **0.90***** | **0.90***** |  |  |  |
| W8 | 0.45** | **0.81***** | **0.84***** | **0.91***** | **0.91***** | **0.99***** |  |  |
| W9 | 0.44** | **0.59***** | **0.63***** | **0.72***** | **0.72***** | **0.85***** | **0.84***** |  |
| (b) |  |  |  |  |  |  |  |  |
| S_above10_ | **0.59***** | **0.50**** | **0.50**** | **0.53***** | **0.53***** | **0.52***** | **0.55***** | 0.34* |
| S_whole10_ | **0.50***** | **0.60***** | **0.57***** | **0.60***** | **0.60**** | **0.58**** | **0.59**** | 0.34* |
| DR_s.whole_ | **0.50***** | **0.57***** | **0.53***** | **0.57***** | **0.57**** | **0.56**** | **0.58**** | 0.35* |
| DR_s.above_ | **0.60***** | **0.53***** | **0.52***** | **0.54***** | **0.55***** | **0.51**** | **0.54**** | 0.34* |
| DR_growth_ | 0.36* | 0.31* | 0.28(*) | 0.32* | 0.32* | 0.27(*) | 0.29(*) | 0.18 |
| RGR_irrigated_ | 0.02 | 0.08 | 0.05 | 0 | 0 | 0.01 | 0 | -0.02 |

**(2**) % individuals without necrosis

|  | W2 | W3 | W4 | W5 | W6 | W7 | W8 | W9 |
| --- | --- | --- | --- | --- | --- | --- | --- | --- |
| (a) |  |  |  |  |  |  |  |  |
| W3 | **0.65***** |  |  |  |  |  |  |  |
| W4 | **0.56***** | **0.89***** |  |  |  |  |  |  |
| W5 | **0.54***** | **0.82***** | **0.91***** |  |  |  |  |  |
| W6 | **0.54***** | **0.81***** | **0.91***** | **0.99***** |  |  |  |  |
| W7 | **0.52***** | **0.73***** | **0.85***** | **0.90***** | **0.90***** |  |  |  |
| W8 | **0.50***** | **0.67***** | **0.78***** | **0.84***** | **0.86***** | **0.95***** |  |  |
| W9 | 0.46** | **0.64***** | **0.75***** | **0.76***** | **0.78***** | **0.88***** | **0.87***** |  |
| (b) |  |  |  |  |  |  |  |  |
| S_above10_ | **0.50***** | **0.71***** | **0.79***** | **0.70***** | **0.69***** | **0.70***** | **0.63***** | **0.58***** |
| S_whole10_ | 0.47** | **0.71***** | **0.73***** | **0.66***** | **0.66***** | **0.68***** | **0.62***** | **0.59***** |
| DR_s.whole_ | 0.47** | **0.69***** | **0.70***** | **0.63***** | **0.62***** | **0.62***** | **0.56***** | **0.55***** |
| DR_s.above_ | 0.47** | **0.70***** | **0.77***** | **0.71***** | **0.70***** | **0.68***** | **0.62***** | **0.57***** |
| DR_growth_ | 0.15 | 0.37* | 0.35* | 0.39* | 0.41** | 0.30(*) | 0.33* | 0.32(*) |
| RGR_irrigated_ | -0.17 | 0.11 | 0.19 | 0.08 | 0.05 | 0.09 | -0.02 | 0.08 |

**(3**) % aboveground survival

|  | W2 | W3 | W4 | W5 | W6 | W7 | W8 | W9 |
| --- | --- | --- | --- | --- | --- | --- | --- | --- |
| (a) |  |  |  |  |  |  |  |  |
| W3 | **0.94***** |  |  |  |  |  |  |  |
| W4 | **0.86***** | **0.90***** |  |  |  |  |  |  |
| W5 | **0.87***** | **0.90***** | **0.99***** |  |  |  |  |  |
| W6 | **0.78***** | **0.81***** | **0.91***** | **0.91***** |  |  |  |  |
| W7 | **0.77***** | **0.80***** | **0.90***** | **0.90***** | **0.98***** |  |  |  |
| W8 | **0.76***** | **0.80***** | **0.87***** | **0.87***** | **0.95***** | **0.95***** |  |  |
| W9 | **0.76***** | **0.80***** | **0.87***** | **0.87***** | **0.95***** | **0.95***** | **0.99***** |  |
| (b) |  |  |  |  |  |  |  |  |
| S_above10_ | **0.69***** | **0.72***** | **0.76***** | **0.77***** | **0.85***** | **0.84***** | **0.92***** | **0.92***** |
| S_whole10_ | **0.67***** | **0.70***** | **0.76***** | **0.77***** | **0.82***** | **0.80***** | **0.81***** | **0.81***** |
| DR_s.whole_ | **0.67***** | **0.70***** | **0.76***** | **0.77***** | **0.82***** | **0.81***** | **0.81***** | **0.85***** |
| DR_s.above_ | **0.67***** | **0.69***** | **0.74***** | **0.75***** | **0.84***** | **0.82***** | **0.91***** | **0.91***** |
| DR_growth_ | 0.41** | 0.33* | 0.26 | 0.28 | 0.26 | 0.26 | 0.36* | 0.36* |
| RGR_irrigated_ | -0.01 | 0.09 | 0.23 | 0.20 | 0.31(*) | 0.25 | 0.20 | 0.20 |

**Table S6** Results of analyses across all 42 initial species (i.e., including the species excluded in the main analyses presented in Table 1) for effects of treatment, species and their interaction (treatment x species) on plant performance under the drought. For details see Table 1. The two species excluded (*Medicago lupulina, Trifolium repens*) were forbs and therefore the results from grasses presented here are the same as those presented in Table 1. Note that all results are qualitatively the same as in Table 1.

|  | **Treatment** | **Species** | **Treatment**  **x species** |
| --- | --- | --- | --- |
| ***All species*** | |  |  |
| Whole-plant survival | 32.45*** | 203.81*** | 21.72 |
| Aboveground survival | 50.73*** | 293.20*** | 39.17 |
| Relative growth rate | 6.80** | 2.54*** | 0.96 |
| ***Grasses*** |  |  |  |
| Whole-plant survival | 16.20** | 74.71*** | 9.37 |
| Aboveground survival | 27.72*** | 124.86*** | 19.75 |
| Relative growth rate | 11.27** | 1.22 | 1.20 |
| ***Forbs*** |  |  |  |
| Whole-plant survival | 3.69 | 45.97** | 1.50 |
| Aboveground survival | 20.69** | 88.54*** | 5.82 |
| Relative growth rate | 3.79(*) | 3.45*** | 0.80 |

**Table S7** Drought effects on survival (whole-plant and aboveground) and relative growth rate within individual species (compare Fig. 3a, b). Generalized mixed models (for survival) and linear mixed model (for growth) with treatment as a fixed effect and block as a random effect were run for each species. Where all individuals survived in both treatments, no values are given for survival. The treatment effect on survival and growth is given as Chi^2^ and F, respectively. Significant effects are marked bold, and significance levels are (*) *P* < 0.1, * *P* < 0.05, ** *P* < 0.01, *** *P* < 0.001. For species codes, see Table S1. Grasses and forbs are indicated with G and F, respectively.

| **Species** | **Whole-plant survival** | **Aboveground survival** | **Relative growth rate** |
| --- | --- | --- | --- |
| ACHIMI (F) | - | - | 1.89 |
| AGROCA (G) | **4.03*** | **6.17*** | 1.75 |
| AGROST (G) | <0.001 | 0.23 | 0.001 |
| ALOPPR (G) | 0.86 | **4.90*** | 3.51(*) |
| ANTHOD (G) | <0.001 | 0.24 | 0.22 |
| ARRHEL (G) | 0.32 | **5.74*** | 2.07 |
| BRACPI (G) | 0.4 | 1.24 | 1.04 |
| BRIZME (G) | 1.73 | **5.68*** | 0.18 |
| CENTJA (F) | 0.23 | 0.23 | 0.08 |
| CERAHO (F) | **6.41**** | **9.53**** | **9.81**** |
| CIRSOL (F) | <0.001 | <0.001 | 0.03 |
| CREPBI (F) | <0.001 | 0.33 | 1.3 |
| DACTGL (G) | <0.001 | 0.33 | **6.13*** |
| DAUCCA (F) | - | <0.001 | 0.59 |
| ELYMRE (G) | 0.25 | **4.03*** | **5.99*** |
| FESTGU (G) | 0.34 | 0.96 | **2.72(*)** |
| FESTOV (G) | - | - | 0.45 |
| FESTPR (G) | - | 0.23 | 0.28 |
| FESTRU (G) | - | 0.27 | 1.27 |
| GALIMO (F) | - | <0.001 | 0.45 |
| HELIPU (G) | - | <0.001 | **6.06*** |
| HOLCLA (F) | <0.001 | <0.001 | 1.33 |
| LATHPR (F) | **4.34*** | **5.17*** | 0.99 |
| LEONHI (F) | 0.002 | 0.40 | 0.09 |
| LEUCVU (F) | - | - | 0.39 |
| LOLIPE (G) | - | <0.001 | **15.86***** |
| LOTUCO (F) | - | <0.001 | **5.33*** |
| PHLEPR (G) | **3.89*** | **5.10*** | **4.67*** |
| PLANLA (F) | - | - | 3.00(*) |
| POAPR (G) | 3.28(*) | 3.66(*) | **4.75*** |
| POATR (G) | **9.26**** | **6.97**** | **6.68**** |
| PRUNVU (F) | 0.46 | 3.57(*) | **4.32*** |
| RANUAC (F) | 3.76(*) | **5.87*** | **4.26*** |
| RANUBU (F) | 0.16 | 0.94 | 1.59 |
| RANURE (F) | 0.002 | 2.95(*) | **11.5***** |
| RUMEAC (F) | 0.002 | 0.002 | 2.03 |
| RUMECR (F) | <0.001 | <0.001 | 0.05 |
| TARARU (F) | - | 1.48 | 1.86 |
| TRISFL (G) | 3.58(*) | **6.01*** | **20.81***** |
| VICICR (F) | **6.09**** | **7.46**** | 2.31 |
| MEDILU (F) | 1.57 | 1.03 | 0.70 |
| TRIFRE (F) | **19.54***** | **18.53***** | 0.001 |

**Figure S4** **(a)** Drought resistance of survival (DR_s.whole_, response ratio of whole plant survival in the dry treatment relative to the irrigated treatment) and **(b)** drought resistance of growth (DR_growth_, a ratio of median RGR in the dry relative to the irrigated treatment, compare Fig. 3). Values below the line at 0.7 indicate a decrease in RGR in the dry relative to the irrigated treatment. Species are sorted by increasing drought resistance. Grasses and forbs are indicated with G and F, respectively. For species codes, see Table S1.

**Table S8** Compilation of relations of performance parameters over 40 species (compare Fig. 4), and over 42 species including the two species excluded from the main analyses (MEDILU, TRIFRE both forbs). All analyses were conducted combining both life forms, and separately for grasses and forbs. Parameters are drought resistance based on whole-plant survival (DR_s.whole_), aboveground survival (DR_s.above_) and relative growth rate (DR_growth_), relative growth rate at the dry treatment (RGR_dry_) and at the irrigated treatment (RGR_irrigated_,), whole-plant drought survival (S_whole10_). Given are regression coefficients (R^2^), the p-values (*P*) and sample sizes (n). ‘n.s.’ indicates not significant at *P* > 0.1. Note that all results are qualitatively the same between the analyses of 40 vs. 42 species.

|  |  | **40 species** | | | **42 species** | | | |
| --- | --- | --- | --- | --- | --- | --- | --- | --- |
| **Relation** |  | **R^2^** | ***P*** | **n** | **R^2^** | ***P*** | **n** |  |
| RGR_irrigated_ vs. DR_s.whole_ | All | 0.01 | n.s. | 40 | 0.06 | n.s. | 42 |  |
|  | Grasses | 0.04 | n.s. | 19 | -^1^ | - | - |  |
|  | Forbs | 0.14 | 0.099 | 21 | 0.20 | 0.033 | 23 |  |
| RGR_irrigated_ vs. DR_s.above_ | All | <0.01 | n.s. | 40 | 0.03 | n.s. | 42 |  |
|  | Grasses | 0.01 | n.s. | 19 | - | - | - |  |
|  | Forbs | 0.05 | n.s. | 21 | 0.12 | n.s. | 23 |  |
| RGR_irrigated_ vs. S_whole10_ | All | 0.01 | n.s. | 40 | 0.09 | 0.057 | 42 |  |
|  | Grasses | 0.02 | n.s. | 19 | - | - | - |  |
|  | Forbs | 0.16 | 0.077 | 21 | 0.24 | 0.018 | 23 |  |

^1^The results for 40 and 42 species are the same for grasses since only forbs were excluded from the main analyses.

**Figure S5** Relation between species’ drought resistance based on whole-plant survival (DR_s.whole_; a, c, and e), and growth (DR_growth_; b, d, and f) and their annual rainfall niche based on the 5^th^ percentile (i.e., the dry end; a and b), mean (c and d), and the 95^th^ percentile (i.e., the wet end; e and f). Relationships were not significant (*P* > 0.1). The stippled area in (a) illustrates that no drought-sensitive species exhibited rainfall niches ranging into dry areas (i.e., no low 5^th^ percentiles).

**Reference**

Ellenberg H, Weber HE, Düll R, Wirth V, Werner W, Paulißen D (1991) Zeigerwerte von Pflanzen in Mitteleuropa. Scripta Geobot 18:1–260

Engelbrecht BMJ, Kursar TA (2003) Comparative drought-resistance of seedlings of 28 species of co-occurring tropical woody plants. Oecologia 136:383–393

IRRI (1996) Standard evaluation system for rice, 5^th^ edn. International Rice Research Institute, Manila
